# Platelets, Thrombo-Inflammation, and Cancer: Collaborating With the Enemy

**DOI:** 10.3389/fimmu.2019.01805

**Published:** 2019-07-31

**Authors:** Ana Luisa Palacios-Acedo, Diane Mège, Lydie Crescence, Françoise Dignat-George, Christophe Dubois, Laurence Panicot-Dubois

**Affiliations:** ^1^Aix Marseille Univ, INSERM 1263, INRA 1260, Center for CardioVascular and Nutrition Research (C2VN), Marseille, France; ^2^Department of Digestive Surgery, Timone University Hospital, Marseille, France

**Keywords:** platelets, thrombosis, inflammation, cancer, NETs

## Abstract

Platelets are small anucleate cells present in the blood stream, their typical role in primary hemostasis has been well-described. However, new evidence suggests that they have critically important roles in cancer progression and inflammation. Cancer cells can activate platelets, thus using them as physical shields from blood shear forces and natural killer (NK) cells. The activated platelets may also regulate hematopoietic and immune cell migration toward the tumor site; therefore, contributing to the cancer-associated inflammation. The activation of platelets by cancer cells may also contribute to metastasis and cancer progression by stimulating deep venous thrombosis and neutrophil extracellular trap formations (NETs) that “hide” cancer cells. We strived to review the current literature to dissect the role of platelets in cancer-associated thrombosis and tumor microenvironment inflammation.

## Platelet Morphology and Physiology

Platelets were first described by Bizzozero in 1882 who described them microscopically and established that platelets were the first component of the blood to adhere to damaged blood vessel walls *in vivo* and, *in vitro* (1). Since his discovery, platelets have been traditionally linked to hemostasis and thrombus formation (1–4). However, recent studies have shown that they are key players in tumor progression and metastasis, inflammation, atherogenesis, and antimicrobial host defense (1–4).

Platelets are arguably the most beautiful cells in the human body as they have an extraordinary capacity for morphological change and powerful secretion properties (5). As small, anucleate, discoid cells they are the smallest in blood circulation; measuring 2–5 μm in diameter with a thickness of 0.5 μm and a mean cell volume of 6–10 fl (5–7). Platelets are originated from big, nucleated cells called megakaryocytes that reside in the bone marrow and are part of the hemopoietic cell line (6). Platelets have an average lifespan of 5–7 days in the blood stream; where they endure such harsh conditions that as they age, they are reduced in size (5, 8). The average healthy human has 150,000–400,000 platelets per microliter (platelets/μl) in circulation at any given time, and changes in total platelet count and mean platelet volume are often related to pathological conditions and are used as an acute inflammatory marker (5, 9, 10).

The platelet membrane is covered in glycoproteins like GPIbβ-IX-V, GPVI, and GPαIIbβIII; which are essential for complete platelet aggregation and adhesion (11). The membrane also has protease activated receptors: PAR-1, PAR-4, and the P2Y family receptors that mediate activation and aggregation (11). Platelets also contain three different kind of secretory granules: α-granules, dense granules, and lysosomal granules (12, 13).

The α-granules are the most represented and contain membrane-associated and soluble proteins that are expressed in the platelet membrane when it is activated (13). These membrane markers are involved in various processes; including cell adhesion, coagulation, inflammation, cell growth, and host defense (5, 13). They include P-selectin, fibrinogen, vonWillenbrand factor, epidermal growth factor, vascular endothelium growth factor, platelet-derived growth factor, and complement C3 and C4 precursors; to name a few (3, 7, 10).

Dense granules, on the other hand, are slightly rarer with just three to eight per human platelet (7, 13). They contain high concentrations of adenine nucleotides, specifically ADP and ATP; along with serotonin and histamine, which are released upon platelet activation (7, 13, 14). The third granule group, or lysosomes, is the least common with only 1–2 per cell (7, 13). They contain protein degrading enzymes like cathepsins, elastases, collagenases, and glucosidases as well as LAMP-1, LAMP-2, and CD63 (7, 13, 14).

Platelet activation may occur through contact with different agonists, the most predominant being: thrombin, ADP, von Willenbrand factor, and collagen (15). Thrombin is the most powerful platelet agonist; it acts on the GPIb-IX-V and the PAR receptors (7, 15). The PAR receptors have 4 subgroups: PAR2 is not present in the platelets and PAR3 functions only as a cofactor to PAR4 activation (15, 16). PAR1 is the most potent receptor for thrombin induced platelet activation, as it more sensitive to lower thrombin levels than PAR4 (15, 16).

Another strong platelet activator is adenosine diphosphate (ADP); it can be exogenous or released from the dense granules of activated platelets themselves; constituting an activation loop between converging platelets (10, 17). The ADP receptors in the platelets are the P2Y protein family (17). P2Y1 initiates ADP-induced platelet aggregation and is responsible for platelet shape change and P2Y12 amplifies and stabilizes the aggregation response (17). As the alpha granules contain ADP, this can constitute an activation loop between platelets that amplifies their aggregation (2, 17).

Von Willenbrand factor (vWF) is a large glycoprotein produced in the Weibel-Palade body of endothelial cells and by megakaryocytes; it is present in the platelet alpha granules and subendothelial connective tissue (7, 18, 19). It plays an essential role in primary and secondary hemostasis; as a mediator of platelet adhesion, and as a carrier for coagulation factor FVIII (18). The vWF is exposed in activated endothelial cells where it interacts with platelet GPIbα and supports platelet translocation to the subendothelium (19). The platelet αIIbβIII integrin also interacts with vWF, causing a cross linking of platelets that enables platelet aggregation and plug formation (18, 19).

When platelets have been activated, they expose negatively charged phosphatidylserine (PS) on their membrane through activation of scramblase (e.g., TMEM16F) (20). This acts as an anchor for the assembly of the prothrombinase complex which converts fibrinogen to fibrin (19). Activated platelets also contribute to the intrinsic pathway of coagulation by secreting Poly-P in their dense granules that activates fXII (19). Meanwhile, coagulation in itself will also activate platelets, as thrombin will cleave and activate PARs on the platelets; thus creating a positive feedback loop that greatly amplifies the hemostasis/coagulation process (20, 21).

## Platelets, Thrombosis, and Cancer

### Tumor Cells Can Activate Platelets

The association between cancer and thrombosis has been known since 1865 when Armand Trousseau first described that localized cancers can induce venous thrombus formation at distant sites (21, 22). This malignant-associated thrombosis is one of the most common clinical manifestations in cancer patients and is associated to worse prognosis and survival (23). The major reason for the high thrombotic risk in cancer patients is that cancer cells can activate platelets and stimulate aggregation through direct and indirect mechanisms (19, 21). The tumor-cell induced platelet aggregation (TCIPA) has been demonstrated in various cell lines like pancreatic, colorectal, and kidney (24–26). Additionally, the TCIPA has been correlated to higher metastatic potential (19). There are several mechanisms involved in in platelet activation and TCIPA (20).

An important mechanism of TCIPA is the cancer cell secretion of thrombin (15, 27). Thrombin is a serine protease that converts fibrinogen to fibrin, but also activates coagulation factors V, VIII, XI, and XIII and the PAR receptors on platelets themselves (15, 19). Pancreatic and lung cancer in specific have been proven to activate platelets via thrombin secretion as well as thromboxane A2 secretion (28, 29). Tumor cells also express ADP, which activates platelets via the P2Y1 and P2Y12 receptors, making platelets release more ADP from their dense granules and thus activating other nearby platelets (30, 31). Interestingly; colon, prostate, and breast cancer cells can bind platelet FcγRIIa and induce dense granule secretion in the platelets (11). Different cancer types like squamous and germinal have also been proven to express podoplanin which binds to platelet-expressed CLEC-2 and induces platelet activation (32).

Tissue factor is arguably the main activator of the coagulation cascade once it comes into contact with activated factor VIIa in the blood stream (2, 5, 30). Tissue factor is often expressed in cancer cells and cancer derived microparticles (2, 6, 27). Elevated levels of TF in the serum has been evidenced in several types of cancer and in chemotherapy-induced thrombosis (28). Platelet as well as cancer derived microparticles have also been described to express Tissue Factors in their membrane, and thus contribute to platelet activations and cancer-related thrombosis (33–35).

There are other indirect mechanisms of platelet activation by the cancer cells. For example, cancer-cell expressed mucins can force platelets and granulocytes to interact (36). Subsequently, there is bidirectional signaling and Cathepsin G release by the granulocytes, which cleaves the platelet protease activated receptor-4 (PAR4) and activating G proteins (G_q_ and G_12/13_) to induce shape change and platelet activation (36, 37). There are also malignancy-linked deficiencies of the vWF cleaving protease: ADAM13. Its deficiency causes large vWF multimers to circulate which can in turn activate platelets (11, 36, 38). A correlation between the presence of metastatic tumors and the concentration of vWF multimers in circulation, as well as aberrant ADAM13 in circulation has been previously demonstrated (39, 40).

The activation of platelets by cancer cells has a myriad of pro-cancerous effects like stimulating tumor growth, preparing the metastatic niche, and helping the metastatic cells survive in circulation. The induction of a cyclooxygenase 2 (COX-2) mediated paracrine signaling between the stromal and epithelial cells in the adenoma mediated by activated platelets can give the ensuing cancer cells a more aggressive phenotype (41–43). However, it has been shown that low-dose aspirin can have an antimetastatic effect by inhibiting COX-1 (43–45). This inhibition would decrease the cancer-mediated platelet activation and aggregation; thus, having an anti-metastatic effect on the tumor cells (41, 44).

### Platelets Influence Tumor Growth

Platelets have a myriad of growth factors stored in their alpha (α) granules (5–7, 13). They are present in the tumor microenvironment outside of the vasculature where they can come into direct contact with the malignant cells (31, 46). When activated, they secrete transforming growth factor beta (TGF-β), vascular endothelial growth factor (VEGF), and platelet derived growth factor (PDGF) (47, 48). These factors not only induce tumor growth, but also promote angiogenesis and tumoral neovascularization (14).

It is important to also take into account that platelets also have anti-cancerous effects. Recently, platelet-derived microRNA has been identified as an important regulator of tumor development (49). Platelet-derived microparticles transfers miR-24 into cancer cells which targets mt-Nd2 and Snora75; triggering mitochondrial regulation and inhibiting tumor growth (49). This shows that platelet function and effects on cancer progression may be stage and context dependent (21, 49).

### Platelet Receptors Mediate Distant Pre-metastatic Niche Preparation

Platelets are covered in membrane receptors that promote heterotypic cell interactions (27, 30, 31). These interactions play a crucial role in tumor growth and metastatic spread (11, 30, 31). Cancer cells that enter blood circulation during the metastatic process are exposed to high shear stress and to the immune system; to survive they use activated platelets to shield themselves (10, 27).

P-selectin is expressed on the surface of activated platelets and endothelial cells and is an important adhesion molecule (27). Cancer cells can bind to platelet P-selectin through TCIPA and form aggregates to protect themselves from the blood circulation and “hide” from NK cells (50). It has been proposed that platelet αIIbβ3 integrin can link fibrin with tumor αVβ3 integrin (19, 46, 47). The role of thrombin and integrin signaling is also very important in the platelet-cancer cell bonding mechanism (4, 27, 51). Thrombin increases the mRNA and protein levels of αVβ3 integrin and serves as a ligand to this receptor, it also increases the secretion of vascular-endothelial growth factor (VEGF) in human prostatic cancer cells (51).

Platelets also have an important effect on the preparation of the pre-metastatic niche (31, 52, 53). Primary tumors secrete metastasis-related proteins to the target organ that stimulate the migration of bone marrow-derived cells to create this pre-metastatic niche and stimulate neo-vasculogenesis (52, 53). Platelets have a role in managing the pre-metastatic communications; they secrete CXCL5 and CXCL7 upon contact with tumor cells to recruit granulocytes for the formation of the early metastatic niche (52–54). Activated platelets also release growth factors from their α granules, as well as metalloproteases that contribute to the degradation of the extracellular matrix and the preparation of the aforementioned metastatic niche (13, 54, 55).

### Circulating Tumor Cell Survival and Arrest Is Mediated by Platelets

It is now widely accepted that increased platelet counts enhance cancer's metastatic power; while thrombocytopenia (low platelet count) may hinder the process (56). As previously stated, platelets have many adhesion molecules including integrins (αIIbβ3), selectins (P-selectin), leucine rich glycoproteins (P-selectin glycoprotein ligand -PSGL-1- and GPIb/V/IX), and immunoglobulin superfamily proteins (platelet-endothelial adhesion molecule -PECAM-1) (30, 31). These molecules allow them to form aggregates with cancer cells to protect them from the shear forces that would otherwise destroy their membranes (31, 50). These aggregates also serve to stabilize cancer cell arrest on the endothelial wall (27, 53, 55).

The TCIPA results in platelets coating the cancer cell and thus protecting it from the natural killer (NK) cells in the blood stream (57). They can also impair the NK cell mediated cytolytic/tumorilytic activity by secreting platelet TGF-β (57). The TGF-β impairs NK granule mobilization and interferon-γ secretion by downregulation the NKG2D immunoreceptor (50, 57). Another way that platelets aid the cancer cells escape the immune system is by membrane protein transfer (47, 48). In the midst of the platelet aggregate; the cancer cells can co-express platelet markers as well major histocompatibility (MHC) molecules to further camouflage themselves (58).

Platelets support cancer cell arrest in the same manner as it contributes to leucocyte arrest: by selectin (P-selectin) dependent rolling/tethering and integrin dependent adhesion (αIIbβ3, GP-Ibα, and vWF all contribute to firm adhesion) (21, 22). It is also important to note that many cancer cells express “platelet receptors” like αIIbβ3, αVβ3, or GP-Ibα (31, 58). These receptors not only help cancer cells escape the immune response but also mediate direct cancer-endothelial and cancer-leucocyte interactions that promote cancer cell extravasation and prepare them for the colonization of the target tissue (31, 59).

Another important TCIPA effect on cancer endothelial transmigration is the release of ADP from the activated platelets' dense granules (31, 60, 61). ADP interacts with the endothelial PY2 receptor (P2Y1R), causing endothelial cell junctions to become laxer and enabling cancer cells to pass through more easily (60–62). Serotonin is also contained in the dense granules, and experimental studies have demonstrated that by blocking its receptor metastatic spread was inhibited (31, 63). Cancer patients that tend to have higher than average serotonin levels in the blood have a worse prognosis and survival (63).

## Platelets, Inflammation, and Cancer

Malignant tumors have often been described as wounds that do not heal (64). Two of the most important tumor characteristics are their constitutive angiogenesis and perennial inflammation as well as the fibroblast infiltration and constant stroma regeneration (64). The vasculature in tumors is often fenestrated, facilitating the trans-endothelial transport, and exposing subendothelial factors like collagen and TF (14, 65). As we have previously stated, cancer cells can activate platelets through the various TCIPA mechanisms; with the added effect of the exposed subendothelial procoagulant factors there is a continuous platelet-activation loop (5, 19, 66). The activated platelets release their granule content that modulates the tumor microenvironment, including pro-inflammatory cytokines (67).

The proinflammatory cytokines released by the platelets are powerful recruiters and activators of leucocytes (67). These molecules include CXCL1, CXCL4, CXCL5, CXCL7, CXCL12 (SDF-1), and interleukin-8 (IL8) (67, 68). The CXCL12 chemokine attracts hematopoietic cells to the tumor site, stimulating tumor growth, and angiogenesis (69). Macrophages are also CXCR4 positive cells that are recruited to the tumor site by the platelet-expressed CXCL12 (67). On the other hand, CXCL5, and CXCL7 platelet secretion in distant sites to the primary tumor recruit granulocytes to prepare the pre-metastatic niche (5, 50, 52–54).

The activated platelets also express IL-1β (synthesized in the platelet from pre-mRNA) (70). The IL-1β induces TF expression in endothelial cells and stimulates expression of endothelial-leucocyte adhesion molecules (70). IL-1β also promotes platelet activation in an autocrine manner via the IL-1 receptor (67).

Transforming growth factor β (TGF-β) expressed and secreted by activated platelets in the tumor microenvironment has immunosuppressive properties and aids in the cancer cell escape from immune system recognition (71). TGF-β is also partially responsible for the transformation of the neutrophils toward a pro-tumorigenic phenotype (67).

## Platelets and NET Formation in Cancer

Neutrophils are the body's first line of defense and have been traditionally characterized by two modes of action: pathogen engulfment and anti-microbial substance secretion (72). In recent years a new function has been identified: neutrophil extracellular traps (NETs) (72). The NETs are the result of the neutrophils' chromatin and granular content being expelled from the nucleus to form a web-like structure (67, 72). This structure can physically entrap and kill pathogens (67, 72). There are recent studies that suggest that NETs may also be involved in tumor progression, metastasis, and cancer-associated thrombosis (73).

Platelet TLR4 can trigger NETosis in activated neutrophils; and histones 3 and 4 released during the process can in turn, activate the platelets in a continuous loop (73, 74). The extracellular DNA in the NETs is capable of binding and activating coagulation factor XII as well as activating platelets directly (75). Additionally, activated platelet P-selectin can prime neutrophils through P-selectin glycoprotein ligand-1 (PSGL-1) activation and trigger NET formation (76, 77). These activation routes suggest that NETs are indeed a procoagulant factor as they provide a strong stimulus as well as a scaffold for thrombus formation (78). NETs promote fibrin deposition, recruit red blood cells and enhance platelet activation, and in turn, platelet activation promotes NET formation (76, 78).

Indeed, the link between NET formation and venous thromboembolism has long been established. In a baboon model of occlusion induced iliac thrombosis, researchers demonstrated an increase in circulation of NETs after 48 h that was maintained for 6 consecutive days; along with the presence of DNA markers in the thrombus (74). Another group demonstrated that plasma DNA is elevated in patients with deep vein thrombosis vs. healthy patients (79).

It is interesting to note that neutrophils originated from cancer patients are more prone to NETosis when exposed to PMA than those from healthy patients (80). This may be partially explained by the NET-activating properties of granulocyte colony-stimulating factor (G-CSF), and IL-8; which are locally secreted by tumor cells (67).

It has been proposed that tumor educated platelets may exert a pro-NETosis effect on the tumor-microenvironment neutrophils (80, 81). Cancer cells can allegedly use the NETs to protect themselves from shear stress in the circulation and from the immune system during the metastatic process (67, 81). The NET-induced platelet activation might play an important role in cancer progression, enhancing TCIPA, and the pro-thrombotic state (76). However, further research and information is needed to shed light on the contribution of platelets to the generation of NETs and their involvement in cancer progression.

## Conclusion

**Figure 1:**
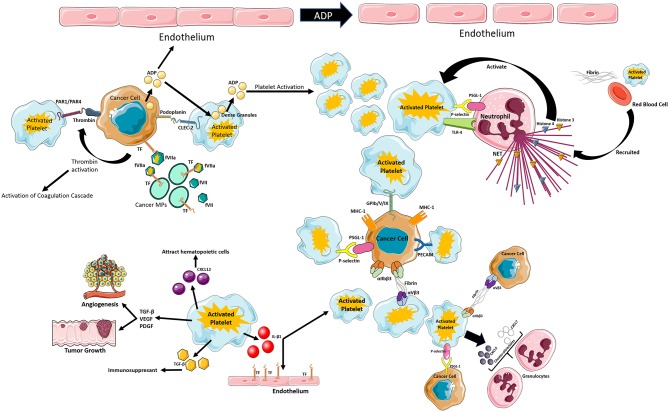
Schematic review of the different mechanisms of platelet activation that can lead to thrombo-inflammation in cancer. Figure created using Servier Medical Art available at http://smart.servier.com/. Copyright Ana Luisa Palacios-Acedo.

Platelets are small but very powerful cells that interact with all components of the circulatory system. They are the main player in primary hemostasis but contribute to the secondary wave as well. As of recently, their involvement in the immune response was described, showing their power in regulating their environment. Their interactions with cancer cells and the tumor microenvironment are very complex and seem to have dual behaviors: pro and anti-cancerous, with the pro-cancerogenic effect out-numbering the anti-cancerous effects (Figure 1). However, it may seem that tumor education of platelets recruits them to the cancer cause, making them an ideal ally of tumor progression. This in turn, causes platelets to be continuously activated enhancing their thrombotic power and augmenting cancer-associated thrombosis. Moreresearch is needed in order to be able to establish the true power of these cells.

## Author Contributions

All authors listed have made a substantial, direct and intellectual contribution to the work, and approved it for publication.

### Conflict of Interest Statement

The authors declare that the research was conducted in the absence of any commercial or financial relationships that could be construed as a potential conflict of interest.

## Abbreviations

ADP: adenosine diphosphate
CLEC 2: C-type Lectin Like 2 receptors
CXCL: chemokine
F: coagulation factor
IL: interleukin
MHC: major histocompatibility complex I
MP: microparticle
NET: neutrophil extracellular traps
PAR: proteinase activated receptor
PDGF: platelet-derived growth factor
PECAM: platelet-endothelial cell adhesion molecule
PSGL-1: P-Selectin protein ligand 1
TF: tissue factor
TGF: transforming growth factor
TLR: toll like receptor
VEGF: vascular endothelial growth factor.
